# Characteristics of Corn Stover Pretreated with Liquid Hot Water and Fed-Batch Semi-Simultaneous Saccharification and Fermentation for Bioethanol Production

**DOI:** 10.1371/journal.pone.0095455

**Published:** 2014-04-24

**Authors:** Xuezhi Li, Jie Lu, Jian Zhao, Yinbo Qu

**Affiliations:** 1 State Key Laboratory of Microbial Technology, Shandong University, Jinan, China; 2 Dalian Polytechnic University, Dalian, China; University of Nottingham, United Kingdom

## Abstract

Corn stover is a promising feedstock for bioethanol production because of its abundant availability in China. To obtain higher ethanol concentration and higher ethanol yield, liquid hot water (LHW) pretreatment and fed-batch semi-simultaneous saccharification and fermentation (S-SSF) were used to enhance the enzymatic digestibility of corn stover and improve bioconversion of cellulose to ethanol. The results show that solid residues from LHW pretreatment of corn stover can be effectively converted into ethanol at severity factors ranging from 3.95 to 4.54, and the highest amount of xylan removed was approximately 89%. The ethanol concentrations of 38.4 g/L and 39.4 g/L as well as ethanol yields of 78.6% and 79.7% at severity factors of 3.95 and 4.54, respectively, were obtained by fed-batch S-SSF in an optimum conditions (initial substrate consistency of 10%, and 6.1% solid residues added into system at the prehydrolysis time of 6 h). The changes in surface morphological structure, specific surface area, pore volume and diameter of corn stover subjected to LHW process were also analyzed for interpreting the possible improvement mechanism.

## Introduction

The high demand for energy worldwide and fossil fuel reserves depletion have generated increasing interest in renewable biofuel sources [1]. The use of bioethanol produced from lignocellulosic material can reduce our dependence on fossil fuels [2]. Lignocellulosic material, for example, waste products from many agricultural activities, is a promising renewable resource for bioethanol production [3]. This generally cheap and abundant material does not compete with food production compared with agricultural crops [4]. The conversion of lignocellulosic material to bioethanol has been a research focus in China for the past decades [5]. In China, corn stover is an agricultural residue that is produced annually. Therefore, research on ethanol production from corn stover is of high importance in the new energy resource development [6]. The conversion process of lignocellulosic material to bioethanol generally includes four steps, namely, pretreatment, enzymatic hydrolysis, fermentation, and distillation [7]. Pretreatment technologies are necessarily applied to lignocellulosic material to decrease recalcitrance and to improve the yield of fermentable sugars [8], [9]. Many pretreatment methods have been proposed and investigated, such as alkaline [10], [11], steam explosion [12], [13], ammonia fiber expansion [14], [15], organic solvent [16], dilute acid [17], [18], and so on. Different pretreatment methods have different mechanisms, for example, they can decrease cellulose crystallinity and/or the polymerization degree, increase accessible surface areas, or selectively remove hemicellulose and lignin from the lignocellulosic material [19]. However, economic and environmental requirements limit the applicability of these methods. An effective pretreatment strategy should also minimize carbohydrate degradation and the production of enzyme inhibitors and toxic products for fermenting microorganisms [20]. One of the most promising pretreatment processes for lignocelluloses material is liquid hot water (LHW) pretreatment [21]–[23]. Some studies have been conducted on the mechanisms of LHW pretreatment [24]–[26]. However, different biomass types have different structures and show different reaction mechanisms.

In the process of ethanol production from lignocellulosic material, enzymatic hydrolysis and fermentation can be performed separately or simultaneously. In separate hydrolysis and fermentation (SHF), these two steps are separate, and SHF can coordinate the inconsistent contradiction between the temperatures for enzymatic hydrolysis and fermentation [27]. In simultaneous saccharification and fermentation (SSF), both steps occur in a single bioreactor where the glucose formed is rapidly converted to ethanol by the yeast. However, solid loading is limited by the higher effective mixing and high viscosity of the system in the SSF process [28]. Semi-SSF (S-SSF) of ethanol production is an operating mode between SSF and SHF. S-SSF consists of two phases, namely, pre-hydrolysis and SSF. To increase substrate concentration, fed-batch S-SSF process was carried out. Fed-batch S-SSF for ethanol production showed that higher substrate concentration and higher ethanol yield can be obtained compared with S-SSF and SSF when a suitable pre-hydrolytic period is selected [29]. In our previous study, LHW pretreatment was applied to corn stover to test the efficiency of enzymatic hydrolysis, and cellulose conversion rates of almost 100% were obtained [30]. In the present work, corn stover samples were subjected to a combination of LHW pretreatment and fed-batch S-SSF to obtain higher ethanol concentration and yield. The effects of different impact factors on the fermentation digestibility of LHW-pretreated corn stover in S-SSF and fed-batch S-SSF are discussed, and the chemical structures and morphological characteristics of corn stover during LHW pretreatment were presented.

## Materials and Methods

### Materials

Corn stover was collected from a corner of field near Jinzhou New District (Dalian, China). It was stated that a permit was not required to collect the corn stover. It was also confirmed that the corn stover is not a protected or endangered species. Corn stover was manually cut into pieces, milled, and screened to collect 20 mesh to 80 mesh fractions. Samples were then homogenized and stored in a plastic bag for subsequent experiments. Corn stover was composed of the following: 10.9% benzene-alcohol (2∶1) extractive, 38.8% glucan, 23.5% xylan, 15.6% acid-insoluble lignin, 2.4% acid-soluble lignin, and 3.7% ash in terms of oven-dried weight. The commercial cellulase used in the study was purchased from Imperial Jade Biotechnology Co., Ltd., Ningxia, China. Cellulase was derived from *Trichoderma longbrachiatum*. *Saccharomyces cerevisiae* was purchased from Angel Yeast Co., Ltd., China. The yeast was activated prior to fermentation. Approximately 1 gram of dry yeast was added to 20 mL of 5% sterilized glucose solution, activated at 38°C for 1 h, cooled to 28°C to 30°C, and used in the fermentation experiment. The fermentation medium contained 0.3% yeast extract, 0.5% peptone, 2.5% KH_2_PO_4_, 0.03% MgCl_2_, and 0.025% CaCl_2_.

### LHW pretreatment

LHW pretreatment was conducted in a 15 L digester with four small tanks (mechanical mill of Shanxi University of Science and Technology, China). Approximately 40 g of corn stover and 800 mL of deionized water were loaded into the small tanks. The start temperature for the pretreatment was 50°C, and the maximum temperature was controlled in the range 170°C to 210°C. The time to maximum temperature was maintained at 100±2 min, and the pretreatment reaction time was set to either 20 or 40 min. Severity factor, which was defined by Overend and Chornet, was used for measuring the pretreatment intensity in LHW. The severity factor provides a way to compare the combined effects of parameters on the changes in the composition to enable a better comparison of results and a better correlation with the compositional changes in the biomass after pretreatment [31]. The severity factors corresponding to different LHW pretreatment conditions are calculated using the following formula (1):
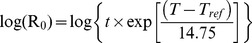
(1)where *t* is the reaction time (min), *T* is the pretreatment temperature (°C), and *T*_ref_ = 100°C.

After pretreatment, the solid residues and the prehydrolysates were separated by filtration with a Bŭchner funnel. The prehydrolysates were analyzed for pH and contents of glucose, xylan, acid-soluble lignin, furfural, and HMF. The solid residues were analyzed for yield and contents of chemical compositions. The solid residues were used for subsequent fermentation.

### S-SSF

For S-SSF process, sample pre-hydrolysis was performed at 50°C for 6 h to 24 h prior to the main SSF phase. The weighted solid residue from LHW pretreatment was added into in 100 mL Erlenmeyer flask that contains pH 4.8 buffers. Cellulase loading was 25 to 50 filter paper unit per gram of oven-dried solid residues. After the pre-hydrolysis time, the medium temperature was adjusted to a constant fermentation temperature and maintained during the subsequent SSF. Then, approximately 1 mL of activated yeast was added into the medium. The fermentation experiments were performed in a constant-temperature incubator for 72 h. The flasks were sealed with rubber stoppers and equipped with syringe needles to remove the generated carbon dioxide. Samples were collected at 0, 12, 24, 36, 48, 60, and 72 h for glucose concentration and ethanol analyses. Glucose and ethanol were determined using the SBA-40D Biological Sensing Analyzer (Biology Institute of the Shandong Academy of Sciences, Jinan, China). Ethanol yield was calculated using the formula (2):
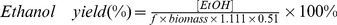
(2)where *[EtOH]* = ethanol concentration at the end of the fermentation minus any ethanol produced from the enzyme and medium (g/L); *f* = cellulose fraction of dry biomass (g/g); *biomass* = dry biomass concentration at the beginning of the fermentation (g/L); 0.51 = conversion factor for glucose to ethanol based on the stoichiometric biochemistry of yeast; and 1.111 = conversion factor of cellulose to equivalent glucose. Each experiment was performed using three parallel samples and the standard error was calculated using Microsoft Excel software in computer.

### Fed-batch S-SSF

Fed-batch was conducted in two ways. The first approach involved the feeding of solid residue (71% moisture content) at the pre-hydrolysis time of 6 h into the fermentation flasks to final substrate concentration of 16.1% (10%+6.1%). The second approach involved the feeding of solid residue in batches at pre-hydrolysis times of 2, 4, 6, and 16 h, into the fermentation flasks. The final solid loadings in the second mode were 17.0%(initial loading of 10%, and 2.8%, 2.3%, and 1.9% at pre-hydrolysis times of 2, 4, and 6 h, respectively) for solid residue abstained at severity factor of 3.95 and 18.7%(10%+2.8%+2.3%+1.9%+1.7%, in which 1.7% was added at pre-hydrolysis time of 16 h) for that at severity factor of 4.54. The other conditions for fed-batch S-SSF were fermentation temperature of 36°C, pH 4.8, cellulase dosage of 40 FPU/g oven-dried solid residues, pre-hydrolysis time of 18 h, pre-hydrolysis temperature of 50°C, and initial solid loading of 10%. The required total cellulase was added before prehydrolysis. Approximately 1 mL of the activated yeast was added into the medium at the beginning of fermentation. The rest of the process steps were similar to those performed in the S-SSF pretreatment of solid residues.

### Analysis

The benzene–alcohol (2∶1) extractive contents were determined using the Chinese National Standard method (GB/T2677.6-1994). The sample was extracted for 6 h with benzene–alcohol mixture (2∶1), then the solvent mixture with extractives was distilled to recover solvent, and remaining residue was dried and weighed for calculating extractives content using formula (3):
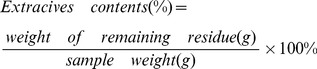
(3)


The content of acid-insoluble lignin (AIL) was determined according to the Chinese National Standard method (GB/T2677.8-1994). Extractives free sample was hydrolyzed with sulfuric acid of 72%±0.1% at 18∼20°C for 2.5 h, then the system was diluted with distilled water to 3% of sulfuric acid concentration, and further hydrolyzed at 100°C for 4 h. After the hydrolysis, the hydrolysis residue was separated by filtration using the filtering crucible, and washed with fresh distilled water to about neutral. Dry the crucible and acid insoluble residue at 105±3°C until a constant weight is achieved for calculating acid-insoluble lignin content, which is a percent of weight of the residue to weight of sample.

The content of acid-soluble lignin (ASL) was determined according to the Chinese National Standard method described in GB/T10337-1989. Using the hydrolysis liquor aliquot obtained in assay of acid-insoluble lignin, measure the absorbance of the sample at 205 nm on a UV-Visible spectrophotometer. 3% sulfuric acid was used to dilute the sample, and the same solvent was used as a blank. The amount of acid soluble lignin was calculated using formula (4)
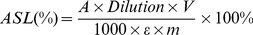
(4)where: A = absorption value at 205 nm; Dilution = dilution factor; V = filtrate volume (ml); ε = absorptivity of biomass [L/(g.cm)]; and m = weight of oven dry sample (g)

The glucan content and xylan content in solid biomass sample were determined according to National Renewable Energy methods. The glucan content and xylan content were calculated using formula (5) and formula (6) respectively:
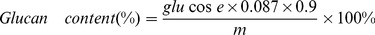
(5)

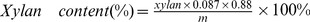
(6)Where: glucose/xylan = glucose/xylan concentration (g/L); m = mass of oven-dried solid residues (g); 0.087 = volume of acid hydrolysis liquid (L); and 0.9/0.88 = conversion factor for glucose to glucan or xylose to xylan.

For the compositions of prehydrolysates, the contents of furfural, HMF, glucose and xylose were determined using HPLC and acid soluble lignin content was analyzed by ultraviolet-spectroscopy using method above.

### Scanning electron microscopy (SEM) analysis

The pretreated samples were washed with deionized water and then dried at 105°C for 4 h. The samples were then coated with gold in a Balzers SCD004 sputter coater and examined in a JEOL JSM-6460 LV SEM (Akishima, Japan).

### Specific surface area, pore size, and distribution analyses

The specific surface area and the pore size of samples were determined by a high-speed automatic surface area and pore size analyzer (NOVA2200e, Quantachrome Instruments Co., USA).

### FT-IR analysis

FT-IR analysis was performed on both the untreated corn stover and the solid residues pretreated at 190°C and 210°C. All samples were dried and pressed into a KBr disc. IR spectra were obtained using a Spectrum One-B FT-IR spectrometer (PerkinElmer, USA) with a resolution of 0.5 cm^−1^ in the range 4000 cm^−1^ to 450 cm^−1^.

## Results and Discussion

### Chemical composition of prehydrolysates and solid residues from LHW pretreatment

The chemical compositions of the prehydrolysates and solid residues from LHW pretreatment at the different temperatures and times assayed are presented in Table 1. Log (R_0_) was the severity factor used to represent pretreatment severity. In the LHW pretreatment, a fraction of the lignocellulosic material was removed from the solid corn stover and transferred to the prehydrolysate. Approximately 24% to 45% of the original material was solubilized according to different pretreatment severity, which resulted in decreased solid residues yield. Acid-insoluble lignin increased with increasing severity factor for severity factors <3.66 because of the quick removal of xylan at lower severity factors. When the severity factor increased to 4.25, the acid-insoluble lignin content decreased and was even lower than the content of untreated raw materials. When the intensity factor was higher than 4.54, the acid-insoluble lignin content increased again. This result may be caused by the formation of “lignin-like” structures obtained as a result of condensation reactions between lignin and carbohydrate degradation products. The condensation substrate was adsorbed on the surface of solid residues, which increased the acid-insoluble lignin. The acetyl groups coupled with xylan were released as acetic acid in the prehydrolysates under the high severity factor, resulting in the decreased pH of the prehydrolysates. The decreased pH resulted in a decrease of the acid-soluble lignin amount of the solid residue, whereas the acid-soluble lignin amount in the prehydrolysates increased. Xylan (including xylose) and glucose were the main two sugars in the prehydrolysates. The xylan content in the prehydrolysates rose progressively with increasing severity factor (>3.95), and then a decrease was detected. This reduction was caused by xylan degradation at high severity factors. During LHW pretreatment, the sugar degradation products that are released into the prehydrolysate, for example, furfural and HMF, inhibit both yeast [32], [33] and enzymes [34]. In previous studies [30], the cellulose conversion rate in enzymatic hydrolysis was high at the severity factor of 3.95, whereas the cellulose conversion rate did not increase significantly with increased severity factor. Therefore, in the present work, the solid residues at the severity factor of 3.95 were selected as the substrates of subsequent fermentation because the degradation products in the prehydrolysates were lower at the pretreatment severity. For comparison, the solid residues at the severity factor of 4.54 were also used as the substrate.

**Table 1:** Chemical compositions of the prehydrolysates and solid residues obtained from LHW pretreatment of corn stover.

Pretreatment conditions		log (R_0_)	Prehydrolysate (g/L)						Solid residues (%)**					
time (min)	temperature (°C)		pH	Xylan*	Glucose	ASL	Furfural	HMF	Yield	Xylan	Glucan	AIL	ASL	Extractives
20	170	3.36	4.19	2.09±0.13	1.21±0.05	1.44±0.05	0	0.10±0.03	76.4	26.02±0.16	42.33±0.03	17.14±0.07	1.7	8.38±0.10
20	180	3.66	3.95	3.09±0.16	1.56±0.03	1.58±0.02	0	0.11±0.03	67.7	15.29±0.14	46.03±0.04	17.57±0.03	1.2	11.23±0.09
20	190	3.95	3.64	5.29±0.15	2.46±0.02	2.07±0.04	0	0.73±0.02	59.6	9.23±0.15	53.67±0.02	15.3±0.04	1.1	15.83±0.06
20	200	4.25	3.37	2.10±0.12	3.54±0.04	2.76±0.05	0.32±0.04	1.96±0.04	57.5	5.23±0.13	53.31±0.02	15.48±0.06	0.5	18.24±0.10
20	210	4.54	3.31	0.55±0.13	4.16±0.01	2.72±0.03	0.36±0.06	2.46±0.03	57.2	5.10±0.12	54.33±0.03	16.56±0.03	0.5	19.78±0.07
40	170	3.66	3.98	3.65±0.11	1.50±0.04	1.53±0.04	0	0.13±0.02	70.3	17.13±0.17	47.42±0.04	18.11±0.05	1.5	8.07±0.08
40	180	3.96	3.81	4.22±0.12	1.98±0.05	1.97±0.02	0	1.16±0.04	62.2	10.62±0.19	50.16±0.03	17.66±0.07	1.1	12.17±0.08
40	190	4.25	3.45	2.55±0.15	3.06±0.03	1.31±0.04	0.29±0.09	1.77±0.01	58.0	5.43±0.12	54.60±0.02	15.42±0.05	0.8	17.85±0.09
40	200	4.55	3.34	0.65±0.16	3.72±0.03	1.63±0.05	0.27±0.02	2.75±0.04	56.0	4.82±0.16	54.73±0.04	17.17±0.04	0.3	19.25±0.07
40	210	4.84	3.34	0.47±0.17	4.32±0.05	1.68±0.02	0.16±0.05	2.51±0.04	55.7	4.53±0.18	54.78±0.03	20.09±0.06	0.3	22.40±0.10

*Based on all of xylose and xylan in prehydrolysate.

**Based on oven dry weight of solid residue, except yield that on basis of weight of untreated corn stover.

### S-SSF

In the work, *Saccharomyces cerevisiae* was used in S-SSF of solid residues obtained from LHW pretreatment of corn stover. In a previous work, *S. cerevisiae* exhibited stable viability and high fermentation efficiency in SHF and SSF [35], [36].

#### 1. Medium

**Figure 1:**
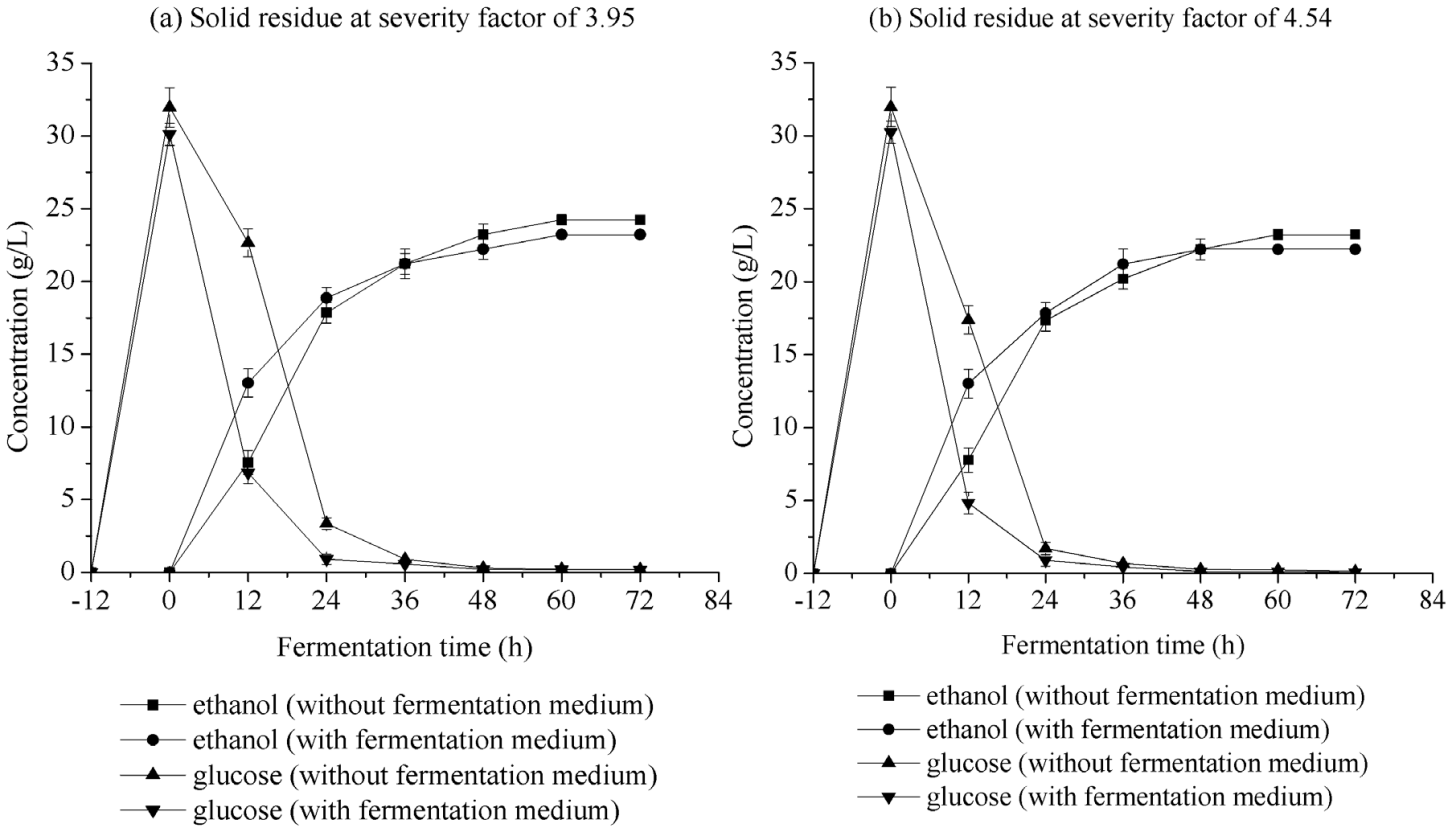
Concentration of ethanol and glucose obtained with and without fermentation medium. Other S-SSF conditions were cellulase loading of 50 FPU/g oven-dried solid residues, substrate concentration of 8.5%, pre-hydrolysis temperature of 50°C, pre-hydrolysis time of 12 h, pH 4.8, and fermentation temperature of 36°C.

During ethanol production, the fermentation medium composition affects the fermentation performance of the yeast [37], [38]. Pre-hydrolysis in S-SSF increases the fermentation sugar concentration in the fermentation system before yeast addition. The high sugar concentration in the fermentation system increases the osmotic pressure, which has a damaging effect on yeast cells [39]. A report suggested that the required nutrients, such as nitrogen and trace elements, are provided in adequate amounts to obtain high fermentation performance in the high sugar concentration medium using *S. cerevisiae* [40]. To obtain efficient ethanol fermentation with *S. cerevisiae*, numerous nutrients are required. Chemicals contribute significantly to the cost of large-scale production. On a laboratory scale, media are often supplemented with peptone and yeast extract. Magnesium, calcium, potassium, and phosphorus that influence the sugar conversion rate are required for the fermentation [41], [42]. For comparison, S-SSF without added nutrients was performed with the solid residues pretreated with LHW at the severity factors of 3.95 and 4.54. The results are shown in Figures 1a and 1b. The final ethanol concentration in media of the added nutrients did not increase significantly; but initial productivity of the fermentation process increased. The ethanol concentration when nutrients were added was higher than that when no nutrients were added before the fermentation time of 36 h (severity factor of 3.95) and 48 h (severity factor of 4.54). By contrast, the ethanol concentration when nutrients were added was lower than that when no nutrients were added after 36 h (severity factor of 3.95) and 48 h (severity factor of 4.54). This result suggests that additional nutrients are not necessary for ethanol fermentation of the pretreated corn stover with the commercial yeast, which decreases production cost.

#### 2. Fermentation temperature

**Figure 2:**
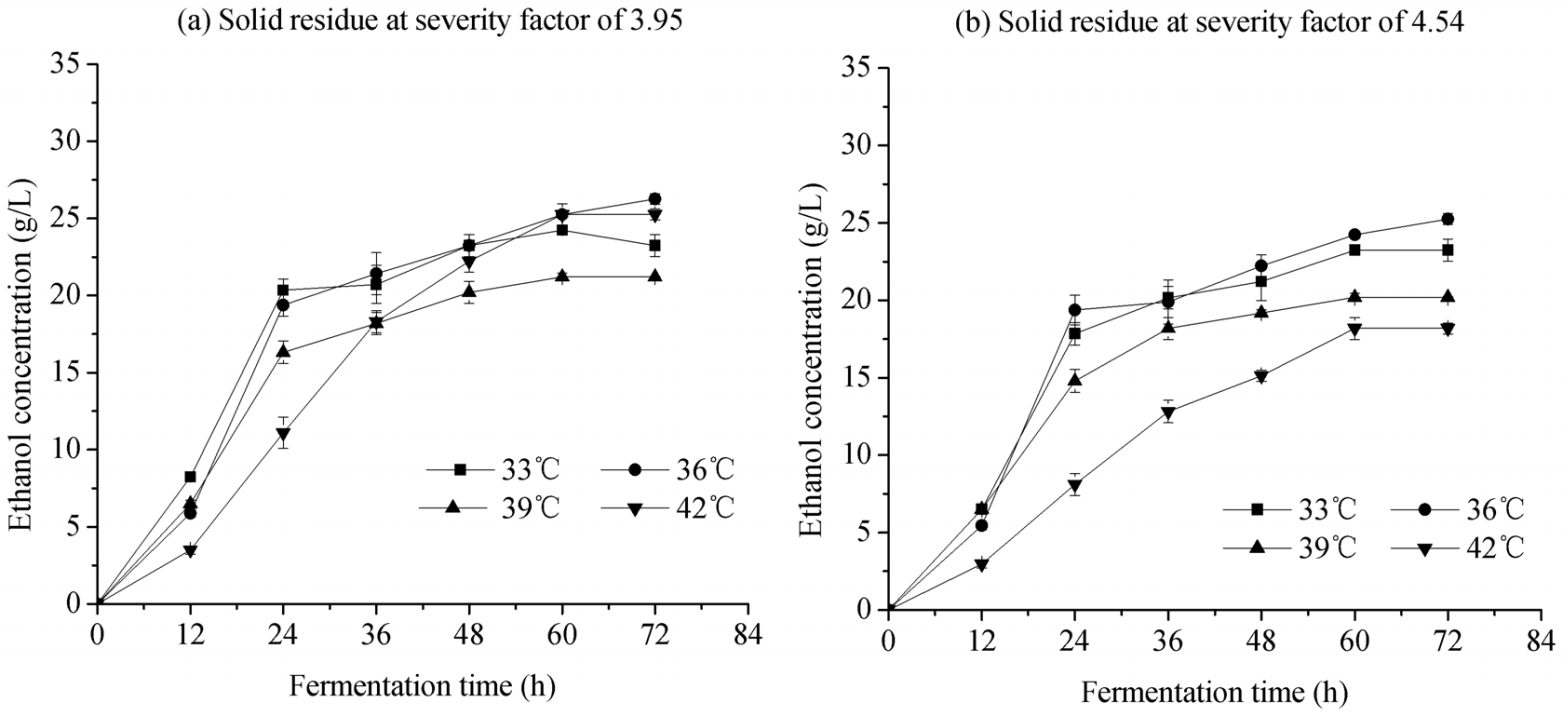
Effect of fermentation temperature on concentration of ethanol and glucose in S-SSF of pretreated corn stover with LHW. S-SSF conditions are same as that in Figure 1 except fermentation temperature.

Fermentation temperature is one of the main technological factors known to impact the activity of *S. cerevisiae* at industrial scale [43]. Optimal fermentation temperature can increase production ethanol yields using *S. cerevisiae*. The impact of fermentation temperature was investigated in S-SSF at 33, 36, 39, and 42°C after 12 h of pre-hydrolysis at 50°C, as shown in Figures 2a and 2b. For the two solid residues from LHW pretreatment of corn stover at the severity factors of 3.95 and 4.54, the highest ethanol yield occurred at temperature of 36°C after 72 h of fermentation time, Thus, S-SSF was performed in the present study at 36°C using *S. cerevisiae*.

#### 3. Pre-hydrolysis time

**Figure 3:**
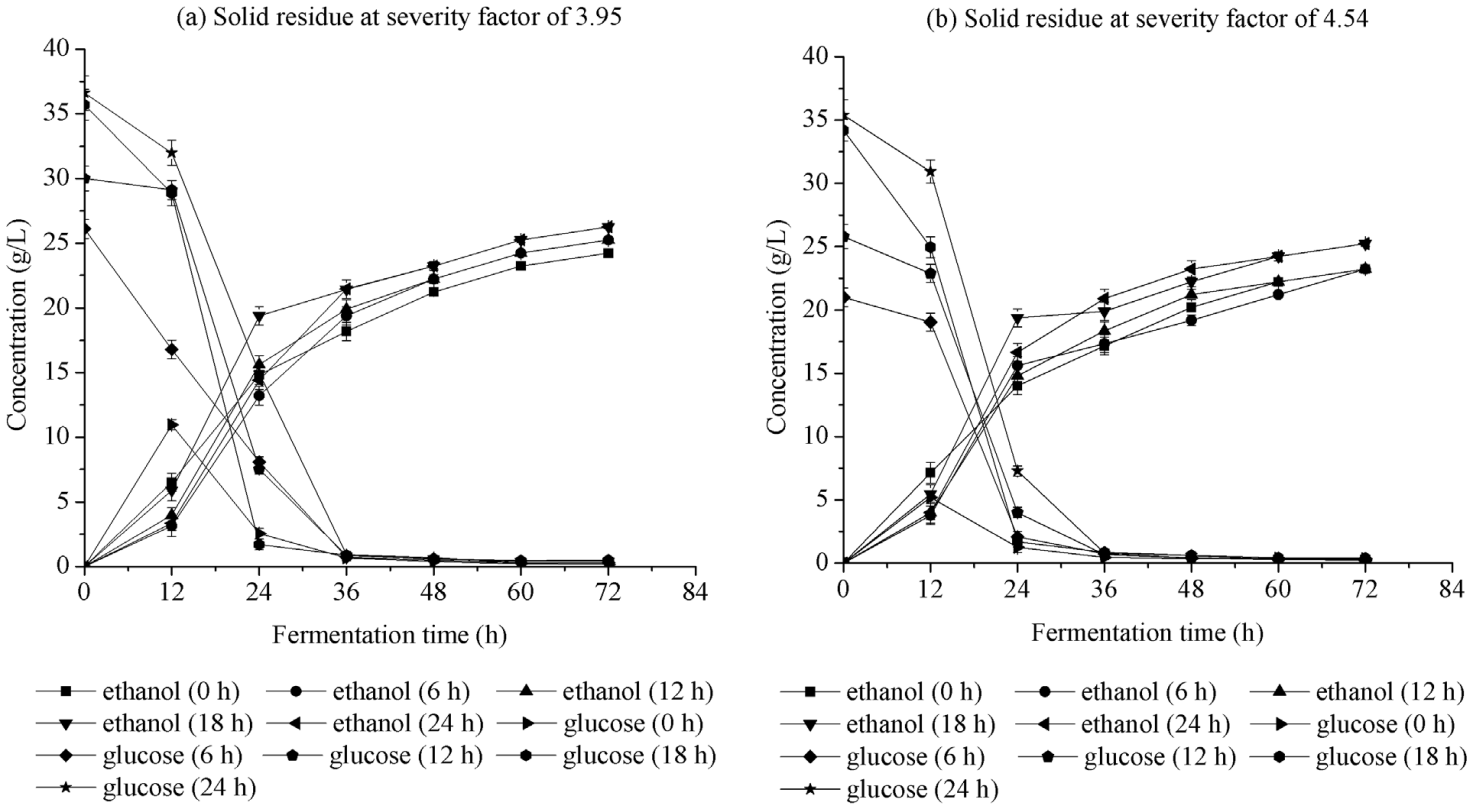
Effect of pre-hydrolysis time onconcentration of ethanol and glucose in S-SSF of pretreated corn stover with LHW. S-SSF conditions are same as that in Figure 1 except pre-hydrolysis time.

The pre-hydrolysis time refers to the initial cellulose hydrolysis for a constant time prior to the main SSF phase. Pre-hydrolysis time, one of the important factors in S-SSF, influenced ethanol concentration. The concentrations of ethanol and glucose with respect to pre-hydrolysis time in S-SSF at 6, 12, 18, and 24 h and the SSF experiments are shown in Figures 3a and 3b. For S-SSF, the initial glucose concentration increased with increasing pre-hydrolysis time. The glucose concentrations gradually decreased from the initial higher values with the extension of fermentation time, which almost approached zero after 36 h. By contrast, the glucose concentration in SSF first increased because of the low ethanol production rate and the high enzyme concentration in the initial period, resulting in glucose accumulation during the initial 12 h. For S-SSF and SSF, the ethanol concentration rapidly increased within the first 24 h, and then increased slowly. This result was due to the exponential growth of yeast because of sufficient substrate supply, and glucose was quickly consumed during this period. As Figures 3a and 3b show, S-SSF at 18 h was different compared with other modes. The glucose concentration was reduced faster and the ethanol concentration increased faster than the other modes at the initial 24 h. The ethanol concentration in S-SSF at 18 h of pre-hydrolysis time is higher than that in S-SSF at 6 h, 12 h, and SSF at the same fermentation time. Ethanol concentration did not increase when pre-hydrolysis time was further extended to 24 h. In S-SSF at 18 h of pre-hydrolysis time, the final ethanol concentration reached 26.3 g/L (severity factor of 3.95) and 25.3 g/L (severity factor of 4.54) at 72 h of fermentation time. Thus, the optimal enzymatic pre-hydrolysis time to obtain the maximum ethanol concentration in S-SSF was 18 h when using pretreated corn stover with LHW as substrate.

#### 4. Enzyme loading

**Figure 4:**
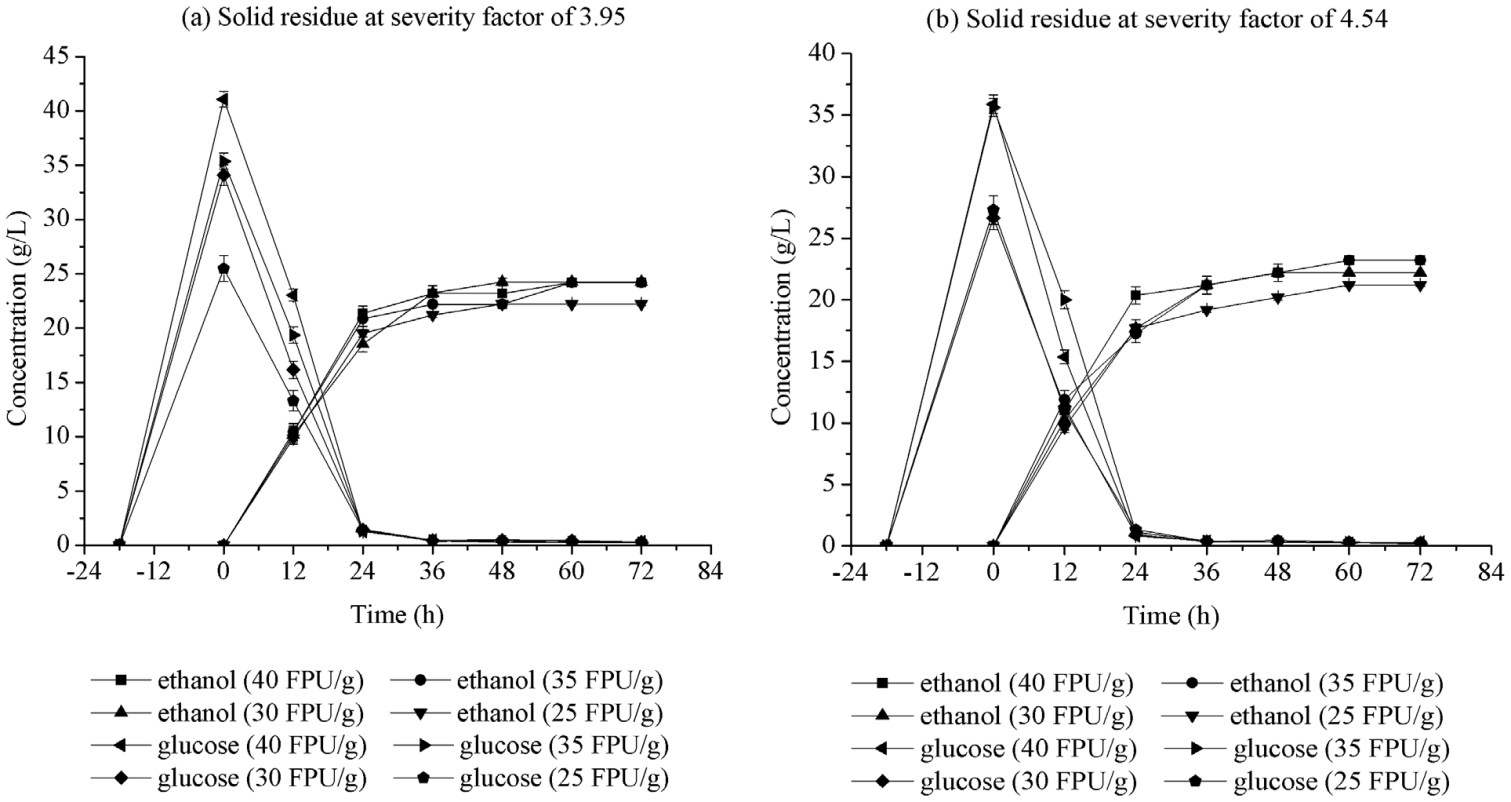
Concentrations of ethanol and glucose produced with different cellulase loadings. Other S-SSF conditions are same as that in Figure 1 except pre-hydrolysis time of 18 h.

Enzyme cost has been recognized as a considerable contributor to bioethanol production cost. Therefore, the fermentation cost can be lowered by decreasing enzyme loading. Theoretically, ethanol yield increases with increasing enzyme loading. However, the complex structure of lignocellulosic material inhibits enzyme activity. From an economic perspective, higher enzyme loadings can result in the waste of a large number of enzymes. The optimization of enzyme loading dosage in S-SSF is a key requirement for large-scale bioethanol production. The results are shown in Figure 4. Ethanol concentrations showed a slight increase with increasing enzyme loading from 25 FPU/g to 30 FPU/g oven-dried solid residues after 72 h. The increase in enzyme loading above 30 FPU/g oven-dried solid residues did not increase ethanol concentration. Residual glucose concentration was less than 0.3 g/L in S-SSF with all enzyme loadings after 24 h. The appropriate cellulase loading was 30 FPU/g oven-dried solid residues.

#### 5. Solid loadings

The interest in high solid loading of enzymatic hydrolysis and fermentation is motivated by reduced liquor volume, resulting in lower operating cost [44]. As a drawback, high solid loading can result in difficulties in stirring the material. In addition, LHW pretreatment resulted in solid residues with higher moisture, complicating the further increase of high solid loading. Therefore, in the present work, S-SSF was carried out at the solid loadings of 8.5%, 10%, and 17% (w/v) of the solid residue from LHW-pretreated corn stover. Table 2 shows the ethanol concentrations and yields obtained from solid residues with LHW pretreated at 190 and 210°C subjected to S-SSF fermentation at different solid loadings. The highest ethanol concentration was obtained with 10% (w/v) solid loading. The ethanol concentration of 33.3 g/L corresponds to 98.4% of ethanol theoretical yield in the pretreated solid residues at the severity factor of 3.95. With the further increase to 17% (w/v) solid loading, the ethanol concentration and yield were even lower.

**Table 2:** Ethanol concentration in S-SSF and Fed-batch S-SSF of solid residues from LHW pretreated corn stover with different solid loadings.

Fermentation methods	S-SSF						Fed-batch S-SSF			
Severity of preteatment of corn stover	Severity factor of 3.95			Severity factor of 4.54			Severity factor of 3.95		Severity factor of 4.54	
Solid loading (%)	8.5	10	17	8.5	10	17	16.1 (10+6.1)	17 (10+2.8+2.3+1.9)	16.1 (10+6.1)	18.7 (10+2.8+2.3+1.9+1.7)
Ethanol concentration (g/L)	26.3±0	33.3±0	21.2±1.0	25.2±0.5	31.3±0.7	31.3±0.5	38.4±0	28.3±1.4	39.4±1.4	39.4±1.4

### Fed-batch S-SSF

As the above analysis shows, S-SSF showed higher ethanol concentration and yield using corn stover pretreated with LHW as substrate. For possible commercial applications, ethanol concentration needs to be further increased to decrease the cost of follow-up distillation. The fed-batch S-SSF experiments results shown in Table 2 indicate that the ethanol concentration of the first feeding mode (feeding one time) were higher than those of the second feeding mode (feeding many times in batches). This difference indicates that the first feeding mode was appropriate for the corn stover pretreated with LHW. Compared with S-SSF, the ethanol concentration increased significantly in fed-batch S-SSF with the first feeding mode. At the same time, the ethanol yield reached almost 80%, which was a better result. In the optimum mode, the ethanol concentrations of 38.4 g/L (severity factor of 3.95) and 39.4 g/L (severity factor of 4.54) as well as ethanol yields of more than 78.6% (severity factor of 3.95) and 79.7% (severity factor of 4.54) were obtained. The optimum conditions for the fed-batch mode should be studied further to improve the ethanol production from corn stover. Table 3 shows a comparison of several ethanol productions using corn stover as the substrate found in the literature and in this work. Compared with the results of other studies, the ethanol concentration reached 39.4 g/L in the current study. The ethanol concentration in this work is higher than those in other studies. However, the high enzyme loadings needed to be reduced in the future studies.

**Table 3:** Comparison of various ethanol productions using pretreated corn stover as the substrate found in the literatures and in the current study.

Pretreated method	Fermentation method	Hydrolysis Temp. (°C)	Temp. in SSF (°C)	Pre-hydrolysis time (h)	Fermentation time (h)	Enzyme loadings	Ethanol concentration (g/L)	Reference
LHW	Fed-batch S-SSF	50	36	18	60	Cellulase 30–35 FPU/g substrate	39.4	In this work
Alkaline	SSF	-	33	-	72	Cellulase 20 FPU/g substrate, β-glucosidase 10 CBU/g substrate	27.8	[58]
Dilute sulfuric acid	SHF	45	35	72	72	Cellulase 15 FPU/g substrate, β-glucosidase 9 CBU/g substrate	30.6	[59]
Dilute phosphoric acid	SHF	45	35	72	48	Cellulase 15 FPU/g substrate, β-glucosidase 9 CBU/g substrate	26.4	[60]
Fungal	SSF	-	37	-	72	Cellulase 10 FPU/g solid	25.0	[61]

### Changes in structure characterization of corn stover after LHW pretreatment

#### 1. Morphological characterization

**Figure 5:**
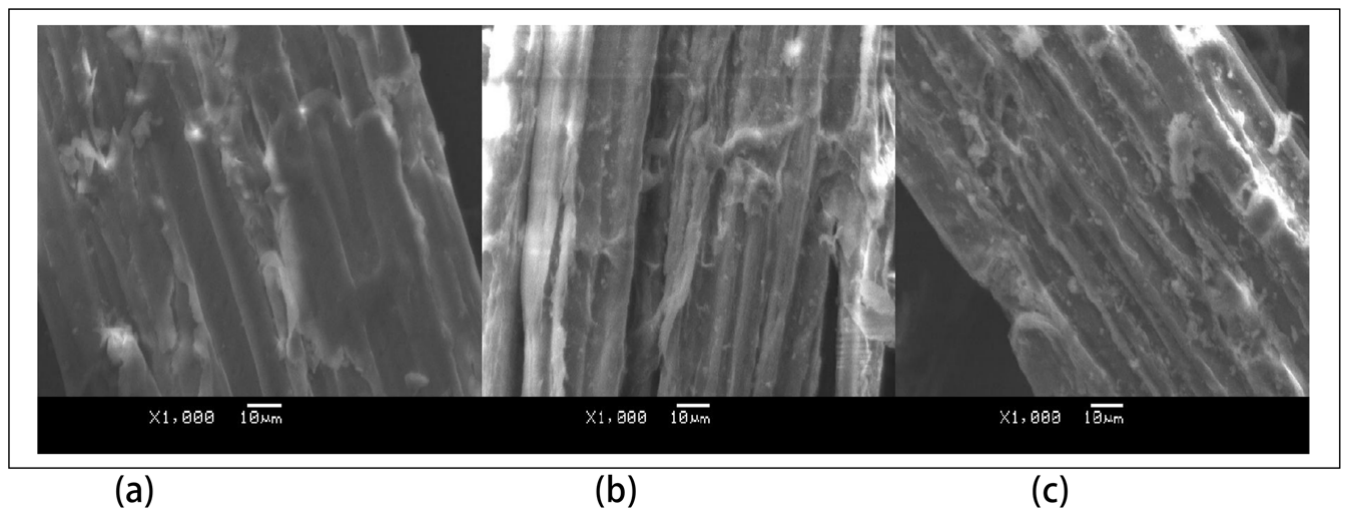
SEM micrographs of untreated and LHW-pretreated corn stover. a: untreated corn stover; b: pretreated corn stover at severity factor of 3.95; c: pretreated at severity factor of 4.54.

SEM was used to observe the changes of morphological characteristics of corn stover before or after LHW pretreatment. The SEM micrographs of untreated and pretreated corn stover are presented in Figure 5. LHW pretreatment significantly disrupted structure of corn stover with increased pretreatment severity and significantly decreased particle size. The surface status was also changed by LHW pretreatment. The untreated corn stover possessed a flat, smooth, rigid, regular, and compact surface structure (Figure 5a). After pretreatment with LHW, the smooth surfaces gradually became rough (Figure 5b and 5c), which was beneficial to reaction with enzyme.

#### 2. Specific surface area, pore volume and pore diameter

As the aforementioned SEM analysis shows, LHW pretreatment could disrupt recalcitrant microstructure significantly to form a smaller average particle size, resulting in small pores in the pretreated sample surface. A greater amount of information related to cellulase action can be obtained from measurements of the pores or “interior” surface area of particles available for penetration by cellulase. In the present work, specific surface area, pore volume, and pore diameter were studied using the Brunauer–Emmett–Teller method by nitrogen adsorption to explain the mechanism involved in enhancing the enzymatic hydrolysis of corn stover with LHW pretreatment. The results are shown in Table 4. The specific surface area of corn stover pretreated with LHW was higher than that of the untreated sample, means that LHW pretreatment led to specific surface area increase. Generally, the specific surface area of particles is inversely proportional to their average particle size. However, in Table 4, the specific surface area decreased when the pretreatment severity factor increased to 4.54. This difference was due to the surface area of the particles being divided into exterior surface area and interior surface area. The specific surface area was affected mainly by the interior surface area. The interior surface area is influenced by pore volume. The pore volume of corn stover at the severity factor of 4.54 was lower than that of pretreated corn stover at the severity factor of 3.95 (Table 4).

**Table 4:** Changes in surface area, pore volume and pore diameter of corn stover before and after LHW pretreatment.

Samples	Untreated corn stover	Pretreated at severity factor of 3.95	Pretreated at severity factor of 4.54
Specific surface area (m^2^/g)	8.55	17.08	10.55
Pore volume (cm^3^/g)	0.001	0.028	0.020
Pore diameter (×10^−9^ m)	2.91	6.77	8.90

Li C et al. reported that the difference of surface area and pore volume between untreated and AFEX treated corn stover was negligible, although SEM tomography have shown large increases in macroporosity after AFEX treatment. But there were a significant increase in the BET surface area (21.6 times greater) and the pore volume (26.6-fold greater) after IL pretreatment [45]. Yoon et al. found that ARP-treatment increased the BET surface area by 50% [46]. Our previous work showed that BET surface area of corn stover increased from 0.329 m^2^/g to 2.878 m^2^/g, about 8.75 times greater, after dilute sulfuric acid pretreatment with acid consistency of 1 g/ml at 170°C for 60 min, and 1∶15 of the ratio of corn stover weight (g) to liquor volume (mL) [47]. In this work, the specific surface area of corn stover increased from 8.55 m^2^/g to 17.08 m^2^/g, which about 2 times greater, after LHW pretreatment at the severity factor of 3.95. The pore volume of pretreated corn stover with LHW increased 28 times compared with that of untreated corn stover. The increased surface area and pore volume provides easier enzyme access to cellulose.

Although the specific surface area of the substrate was provided by the decreased particle size, the pore volume has a significant function in facilitating hydrolysis by cellulase, the interconnecting function of other substrate factors such as pore diameters should also be considered. A report proposed that enzymatic hydrolysis is enhanced when the pore diameter of the substrate is large enough to accommodate both large and small enzyme components to maintain the synergistic action of the cellulase enzyme system [48]. Several extensive studies found that the rate-limiting pore diameter for lignocellulosic substrate hydrolysis was 5.1×10^−9^ m [49]–[53]. The pore diameters of substrates, untreated corn stover, and pretreated corn stover at severity factors of 3.95 and 4.54 were 2.9×10^−9^, 6.8×10^−9^, and 8.9×10^−9^ m, respectively (Table 4). The pore diameters of untreated corn stover were <5.1×10^−9^ m, whereas the pore diameter of pretreated corn stover was >5.1×10^−9^ m. The enlarged pore diameters after pretreatment of LHW enhanced action of enzyme on lignocellulosic substrate, and led to the enzymatic digestibility of corn stover pretreated with LHW increase.

#### 3. FT-IR analysis

**Figure 6:**
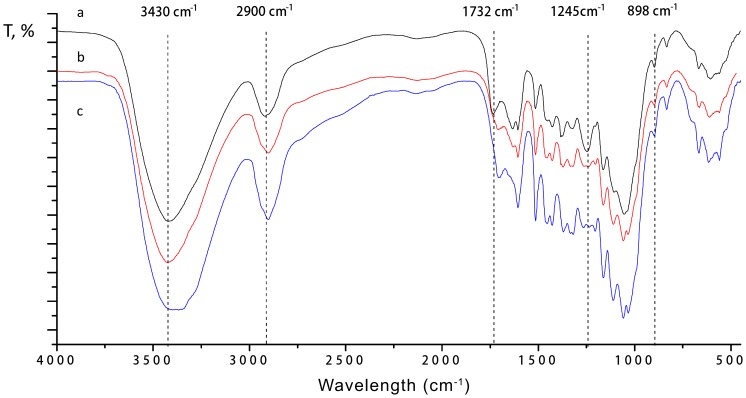
FTIR spectra of untreated and pretreated corn stover at different severity factor. a, b and c denote FTIR spectrum of untreated corn stover, pretreated at a severity factor of 3.95 and pretreated at a severity factor of 4.54, respectively.

FT-IR spectra of the untreated and pretreated corn stover samples are shown in Figure 6. The band at 3430 cm^−1^ is attributed to the O-H stretching of the hydrogen bonds of cellulose [54]. The peak exhibited reduction in intensity, indicating that hydrogen bonds in cellulose were disrupted during LHW pretreatment, and part of the crystalline cellulose in corn stover was disrupted during LHW pretreatment. The band position at 2900 cm^−1^ is attributed to C–H stretching within the methylene of cellulose [55]. The relative absorbance decreased slightly, indicating that the methyl and methylene portions of cellulose were slightly ruptured. LHW pretreatments mostly reduced the intensity of the 1245 cm^−1^ band attributed to the cleavage and/or alterations of acetyl groups, indicating that the acetyl groups were almost completely removed by LHW pretreatment [55]. The ester bond signal at 1732 cm^−1^ was weaker in the spectra of LHW pretreated-samples than that of untreated samples, suggesting that some ester linkages between lignin and carbohydrates were cleaved during LHW pretreatment [56], led to some lignin fractions with low molecular weight partly dissolving out. The chemical compositions described above also shows that a small amount of soluble lignin were detected in the prehydrolysates from LHW pretreatment of corn stover. A small adsorption at 898 cm^−1^ is characteristic of β-glycosidic linkages [57]. The relative adsorption decreased slightly, indicating that pretreatment disrupts the β-glycosidic linkages, led to part of carbohydrates was depolymerized.

## Conclusions

The solid residues after LHW pretreatment of corn stover is suitable to be used as substrate for ethanol production, and the fed-batch S-SSF is one effective process for obtaining higher ethanol concentration and ethanol yield. The optimum feeding process in fed-batch S-SSF of the solid residues was that 6.1% of semi-weighted solid residues at pre-hydrolysis time of 6 h were added into the system. Ethanol concentrations of 38.4 g/L (severity factor of 3.95) and 39.4 g/L (severity factor of 4.54) and ethanol yields of 78.6% (severity factor of 3.95) and 79.7% (severity factor of 4.54) were obtained by the fed-batch S-SSF in the conditions of initial solid loading of 10% and pre-hydrolysis time of 18 h.
